# IL‐10 from plasmacytoid dendritic cells promotes angiogenesis in the early stage of endometriosis

**DOI:** 10.1002/path.5339

**Published:** 2019-10-06

**Authors:** Jau‐Ling Suen, Yu Chang, Yu‐Shiang Shiu, Chia‐Yi Hsu, Pooja Sharma, Chien‐Chih Chiu, Yi‐Ju Chen, Tzyh‐Chyuan Hour, Eing‐Mei Tsai

**Affiliations:** ^1^ Graduate Institute of Medicine College of Medicine, Kaohsiung Medical University Kaohsiung Taiwan; ^2^ Department of Medical Research Kaohsiung Medical University Hospital Kaohsiung Taiwan; ^3^ Department of Obstetrics and Gynecology E‐Da Hospital, I‐Shou University Kaohsiung Taiwan; ^4^ Department of Biochemistry College of Medicine, Kaohsiung Medical University Kaohsiung Taiwan; ^5^ Department of Obstetrics and Gynecology Kaohsiung Medical University Hospital Kaohsiung Taiwan; ^6^ Department of Biotechnology Kaohsiung Medical University Kaohsiung Taiwan; ^7^ Department of Biological Sciences National Sun Yat‐sen University Kaohsiung Taiwan; ^8^ Department of Anatomic Pathology E‐Da Hospital, I‐Shou University Kaohsiung Taiwan

**Keywords:** angiogenesis, endometriosis, IL‐10, plasmacytoid dendritic cell, zebrafish

## Abstract

An elevated level of IL‐10 has been considered a critical factor for the development of endometriosis; however, its detailed mechanism and causal relationship remain unclear. This study explored the cellular source and angiogenic activity of local IL‐10 during the early stage of endometriosis. Using a surgical murine model, we found that localised treatment with exogenous recombinant IL‐10 on the day of surgery significantly enhanced endometriotic lesion growth and angiogenesis, whereas blocking local IL‐10 activity using mAbs significantly suppressed those effects. Adoptive transfer of *Il10* ^+/+^ plasmacytoid dendritic cells into mice significantly enhanced lesion development, whereas *Il10* ^−/−^ plasmacytoid dendritic cells significantly inhibited lesion development. Furthermore, *in vitro* angiogenesis analyses demonstrated that the IL‐10 and IL‐10 receptor pathway stimulated the migratory and tube formation ability of HUVECs as well as ectopic endometrial mesenchymal stem cells through, at least in part, a VEGF‐dependent pathway. We also found that recombinant IL‐10 directly stimulated angiogenesis, based on a Matrigel plug assay as well as a zebrafish model. Pathological results from human endometrioma tissues showed the increased infiltration of CD123^+^ plasmacytoid dendritic cells and higher percentages of cells that express the IL‐10 receptor and CD31 as compared with the corresponding normal counterparts. Taken together, these results show that IL‐10 secreted from local plasmacytoid dendritic cells promotes endometriosis development through pathological angiogenesis during the early disease stage. This study provides a scientific basis for a potential therapeutic strategy targeting the IL‐10—IL‐10 receptor pathway in the endometriotic milieu. © 2019 The Authors. *The Journal of Pathology* published by John Wiley & Sons Ltd on behalf of Pathological Society of Great Britain and Ireland.

## Introduction

Endometriosis is an oestrogen‐dependent and common benign gynaecological disorder. It is associated with chronic inflammation and affects 6–10% of women of reproductive age; however, its aetiology and causative mechanisms remain uncertain 1. The retrograde menstruation/transplantation theory is widely accepted nowadays; that is, the endometrium shed during the menstrual cycle flows from the uterus into the peritoneal cavity 1, 2, 3. The presence of disease in the pelvis has been attributed to lesion growth that results from the following steps, which include the adherence of endometrial cells/tissues to the peritoneum and their subsequent invasion; the establishment of a stable blood supply; and the generation of a chronic inflammatory microenvironment in which retrograde cells or tissues cannot be adequately cleared by the immune response 1, 2, 4, 5, 6, 7. Understanding the underlying mechanism and developing novel therapeutic strategies await further in‐depth studies.

With respect to the chronic inflammatory microenvironment that occurs during endometriosis, a complex cytokine pattern in the peritoneal fluid has been described, including pro‐inflammatory cytokines (IL‐1β, IL‐6, IL‐8, and TNF‐α) as well as the anti‐inflammatory mediator IL‐10 8, 9. It is, however, unclear which factor plays a causative role in the early stage of lesion growth. IL‐10, a well‐known immune‐suppressive cytokine, can be secreted by CD4^+^ T cells 10, B cells 11, and macrophages, as well as by some subsets of dendritic cells (DCs) 12 in response to stimuli. Signalling via IL‐10 and the IL‐10 receptor (IL‐10R) can inhibit antigen‐specific T‐cell activation or differentiation by directly suppressing the function of professional antigen‐presenting cells, such as DCs and macrophages 13. The inhibition of antigen‐presenting cell function and the resulting limitation of T‐cell activation may be the predominant mechanism by which IL‐10 can control immunopathology. The biological function of IL‐10 is pleiotropic, as it has been reported that IL‐10‐conditioned macrophages enhance angiogenesis in age‐related macular degeneration, an eye disease in elderly people 14, 15. However, in addition to immune suppression, the role of IL‐10 in the development of endometriosis has not been well studied, especially its angiogenic activity in the endometriotic microenvironment.

Excessive endometrial angiogenesis has been considered an important mechanism in the pathogenesis of endometriosis 16. Angiogenesis is characterised by the proliferation and migration of endothelial cells, the degradation of extracellular matrix, the formation of tubes as well as loops from pre‐existing blood vessels, and the maturation of the vessel wall 17. High levels of angiogenic factors have been found in the peritoneal fluid in women with endometriosis 18. VEGF, IL‐6, IL‐8, and TNF‐α stimulate the activation of endothelial cells for angiogenesis, suggesting the large extent of cross‐talk between the immune response and angiogenesis in the endometriotic milieu. Understanding the angiogenic mechanism and the mediators involved is critical for the development of an anti‐angiogenic therapy with which to treat endometriosis, particularly recurrent cases.

The role of plasmacytoid dendritic cells (pDCs) in the pathogenesis of endometriosis is largely unknown. Our recent study showed that local high IL‐10 activity significantly promotes the development of endometriotic lesions in a surgical murine model. Interestingly, the major IL‐10‐secreting CD45^+^ cell type in lesions is the pDC (CD11c^+^ PDCA‐1^+^) rather than conventional DCs (CD11c^+^ PDCA‐1^−^) 19. The function of pDCs is well studied in the context of anti‐viral immunity, during which they secrete large amounts of type I IFNs in response to stimuli 20. In addition, pDCs also display tolerogenic phenotypes, which are suggested to promote tumour development, including indoleamine 2,3‐dioxygenase expression in tumour‐draining lymph nodes 21 and induction of regulatory T‐cell expansion in breast cancer 22. Although pDCs may promote tumour development by creating an immune‐suppressive microenvironment, the integrated role of pDCs and IL‐10 in endometriosis awaits further investigation.

In the present study, we hypothesised that IL‐10–IL‐10R signalling may stimulate angiogenesis and in turn promote the growth of endometriotic lesions during the early stage of this disease. We found that IL‐10 secreted from pDCs promoted angiogenesis during the early stage of endometriosis and demonstrate that IL‐10 stimulated angiogenesis through an IL‐10R‐dependent pathway using a HUVEC migration and tube formation assay *in vitro*, as well as an *in vivo* Matrigel plug assay and zebrafish model. In addition, in clinical samples, the frequencies of pDCs, endothelial cell marker CD31, and IL‐10R expression were significantly increased in ectopic endometrioma lesions compared with their corresponding normal counterparts. We also observed the existence of IL‐10^+^ CD123^+^ pDCs in human endometrioma tissues.

## Materials and methods

### Patients

The study was approved by the Kaohsiung Medical University Hospital (KMUH) Institutional Review Board. Participants provided their written informed consent (KMUH‐IRB‐20140187) to participate in this study. Patients of reproductive age (age range 21–49 years) who were treated at the Department of Obstetrics and Gynecology of KMUH were recruited for this project. The stages of endometriosis were determined visually according to the revised American Society of Reproductive Medicine classification 26. Demographic characteristics of the patients with endometrioma (*n* = 10) are shown in supplementary material, Table S1. One ovarian endometriotic tissue sample and one uterine endometrial tissue sample were collected from each patient. Two endometriotic tissue samples were collected from one of the patients.

### Mice and the surgical endometriosis model

The protocol was approved by and adhered to the regulations of the Institutional Animal Care and Use Committee of the Kaohsiung Medical University (Permit Numbers: 103065 and 107 066). Female C57BL/6 mice, NUDE (CAnN.Cg‐*Foxn1* ^*nu*^/CrlNarl), and IL‐10 knockout mice (*Il10* ^−/−^) were maintained by the Animal Center of Kaohsiung Medical University in a pathogen‐free facility. The endometriosis model was established according to our previous study 19. Detailed materials and methods are provided in supplementary material, Supplementary materials and methods.

### pDC purification, and co‐culture of pDCs and apoptotic cells

Detailed materials and methods are provided in supplementary material, Supplementary material and methods.

### Immunofluorescence, immunohistochemistry, and quantitative analysis by the TissueFAXS system

Detailed materials and methods are provided in supplementary material, Supplementary materials and methods, including immunofluorescence for murine lesions with CD31 staining, immunohistochemistry for human endometrial tissues and murine lesions with Ki‐67, and active caspase‐3 staining. Images of the stained tissue were captured by the TissueFAXS imaging system (TissueGnostics, Tarzana, CA, USA) and were then processed using TissueQuest for immunofluorescence and HistoQuest for immunohistochemistry (TissueGnostics).

### Endometrial mesenchymal stem cell (EN‐MSC) culture and conditioned medium (CM)

The procedure used to isolate EN‐MSCs from human uterine eutopic endometrium and ectopic endometriotic lesions has been described previously 23 and is summarised in supplementary material, Supplementary materials and methods. For CM collection, human EN‐MSCs were treated with medium alone, recombinant human IL‐10 (rhIL‐10; Pepro Tech, Rocky Hill, NJ, USA), or mAb against the human IL‐10 receptor (hIL‐10R; eBioscience, San Diego, CA, USA) for 24 h; fresh medium without any treatments was then incubated with the cells for an additional 24 h. Cell‐free CM was then used to culture HUVECs for assessing their angiogenic activity.

### Transwell migration assay and tube formation assay

The method has been described previously 24 and detailed materials and procedures are described in supplementary material, Supplementary materials and methods.

Detailed materials and procedures for transfection of siRNAs and sequences of siRNAs, and western blotting are also presented in supplementary material, Supplementary materials and methods.

### *In vivo* Matrigel plug assay and histological examination

The method has been described previously 25. We used five Matrigel mixtures containing (1) 2 × 10^6^ ectopic EN‐MSCs, (2) ectopic EN‐MSCs mixed with rhIL‐10 (40 U/ml), (3) ectopic EN‐MSCs mixed with rhIL‐10 that had been pre‐incubated with mAb against hIL‐10 (10 µg/ml), (4) recombinant murine IL‐10 (rmIL‐10; 40 U/ml), or (5) rhVEGF (20 ng/ml; R&D Systems, Minneapolis, MN, USA). Detailed materials and procedures are described in supplementary material, Supplementary materials and methods.

### VEGF ELISA

VEGF released into the culture medium was measured using an ELISA kit (R&D Systems).

### Zebrafish model

The methods have been described previously 27. Detailed materials and methods are provided in supplementary material, Supplementary materials and methods.

### Statistical analyses

Student's unpaired *t*‐test was used to examine the continuous variables between groups; the Kruskal–Wallis test followed by Dunn's multiple comparison test for the matched data from a single sample; and Fisher's exact test for zebrafish experiments. Significance was set at *p* < 0.05 for all tests.

## Results

### IL‐10‐expressing pDCs promote endometriosis development in a surgical murine model

**Figure 1:**
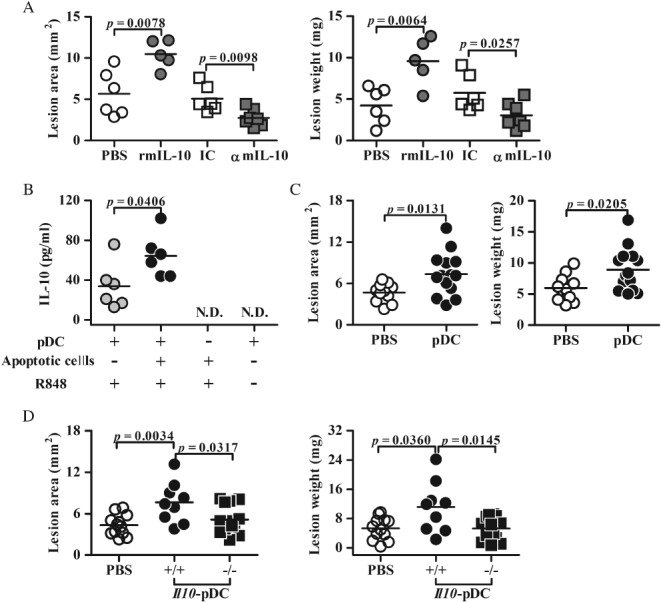
The effect of IL‐10‐expressing pDCs on endometriosis development in a surgically induced model. (A) Lesion area and weight from C57BL/6 mice 4 weeks after surgery. The mice were locally treated at the implantation site as indicated. rmIL‐10, recombinant murine IL‐10; IC, isotype control; αmIL‐10, mAb against mouse IL‐10. *n* = 3 mice in the PBS/rmIL‐10‐treated group; *n* = 4 mice in the IC/αmIL‐10‐treated group. (B) IL‐10 levels from co‐culture supernatants with or without purified splenic pDCs from C57BL/6 mice (*n* = 6), apoptotic cells, and R848. Data from two independent experiments are shown. (C) Lesion area and weight from endometrial surgery mice that received PBS or purified splenic pDCs from syngeneic mice intravenously 1 day before surgery. *n* = 3 mice in the PBS group; *n* = 4 mice in the pDC group. (D) Lesion area and weight from surgery mice that received PBS or purified splenic pDCs from *Il10* ^−/−^ and littermate control (*Il10* ^+/+^) mice intravenously. *n* = 3 mice in the PBS and *Il10* ^*+/+*^‐pDC group**s**; *n* = 4 mice in the *Il10* ^−/−^‐pDC group. The data in A, C, and D represent one of two independent experiments with consistent results. The horizontal line marks the mean for each group. N.D. = not detectable.

To elucidate the role of IL‐10 in the early stage of endometriosis development, exogenous rmIL‐10 or blocking mAb against endogenous mIL‐10 was injected locally under transplanted endometrial tissue on the day of surgery in a murine model. Consistent with our previous finding 19, locally increased IL‐10 levels significantly promoted endometriotic lesion sizes and weights in the mice, whereas locally blocking IL‐10 activity with the mAb significantly inhibited lesion growth (Figure 1A). Thus, dysregulated local IL‐10 activity may enhance the development of endometriosis during the early stage.

As pDCs are the major cellular source of IL‐10 among the infiltrated CD45^+^ immune cells in lesions 19 and infiltrated pDCs made up one of the major immune cell subsets in the lesions (supplementary material, Figure S1), we speculated that pDCs may secrete IL‐10 when they eliminate unwanted apoptotic cells during the menstrual cycle. *In vitro* co‐culture experiments demonstrated that apoptotic cells indeed enhanced the secretion of IL‐10 from TLR7/8‐activated pDCs (Figure 1B). Next, exogenous syngeneic splenic pDCs were intravenously delivered into host mice 1 day before surgery for *in vivo* analysis of the effect of pDCs during the early stage of endometriosis. We observed that increasing pDC numbers by adoptive transfer also enhanced the lesion area and weight in this model (Figure 1C). Furthermore, to elucidate the role of IL‐10‐secreting pDCs, splenic pDCs from either *Il10* ^−/−^ mice or their littermate controls (*Il10* ^+/+^) were transferred intravenously into host mice, which were then used to establish the surgically induced model. We observed that exogenous *Il10* ^+/+^ pDCs significantly promoted lesion growth, but *Il10* ^−/−^ pDCs did not (Figure 1D). These data suggest that infiltrated pDCs can secrete IL‐10 in response to apoptotic cells and consequently promote the growth of endometriotic lesions in the context of endometriosis.

### IL‐10 activity is associated with angiogenesis in the lesion

**Figure 2:**
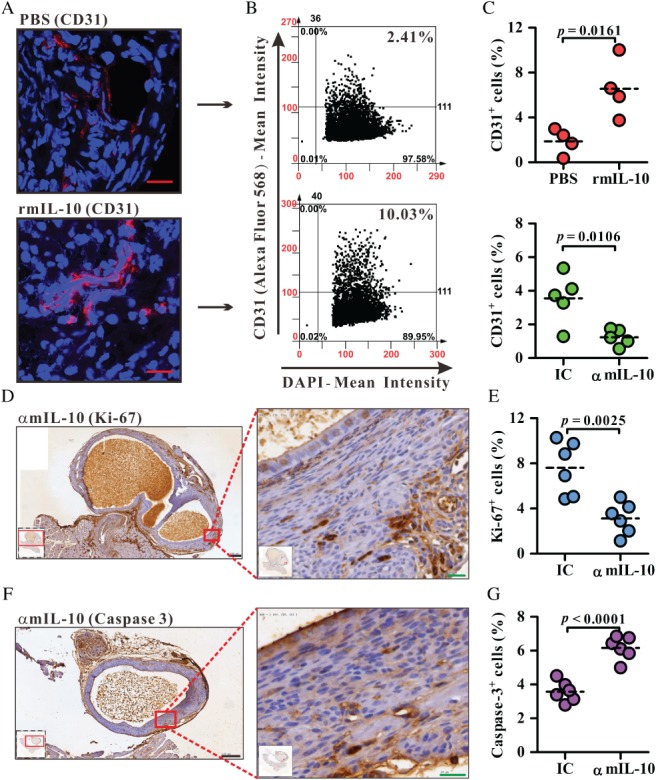
Quantification of CD31^+^ cells in endometriotic lesions in the murine model. (A) Representative immunofluorescence sections of lesions from PBS‐ or rmIL‐10‐treated mice. Blue, nucleus (DAPI); red, CD31 (Alexa Fluor 568). Red scale bars = 20 µm. (B) Representative dot plots analysed by TissueQuest software. (C) The frequency of CD31^+^ cells among DAPI^+^ cells in the whole field of each lesion. IC, isotype control; αmIL‐10, mAb against mouse IL‐10. One dot represents one section from each endometriotic lesion. One endometriotic lesion was sampled per mouse. *n* = 4 mice in the PBS/rmIL‐10‐treated group; *n* = 5 mice in the IC/αmIL‐10‐treated group. (D, E) Representative immunohistochemistry sections of lesions for Ki‐67 or active caspase‐3 (brown colour) from αmIL‐10‐treated mice. Blue, haematoxylin counterstain. Black scale bar (left panels) = 200 µm; green scale bar (right panels) = 20 µm. (F, G) The frequency of Ki‐67^+^ cells or active caspase‐3^+^ cells among haematoxylin^+^ cells in the whole field of each lesion. One dot represents one section from each endometriotic lesion. Two endometriotic lesions per treatment were from one mouse. *n* = 3 mice in the IC/αmIL‐10‐treated group. The horizontal dashed line within the vertical points marks the mean for each group. The data represent one of at least two independent experiments with consistent results.

Next, we explored the effect of IL‐10 on angiogenesis in the lesions, as angiogenesis is a key step for lesion survival and growth during the early stage of endometriosis development 28. We used CD31 as the marker for evaluating the degree of angiogenesis in histological tissue sections 29, 30. Tissue quantification experiments demonstrated that increased local IL‐10 levels significantly enhanced the frequency of CD31^+^ cells in lesion sections, whereas blocking local IL‐10 activity significantly decreased the frequency of CD31^+^ cells in lesion sections (Figure 2A–C). In addition, blocking local IL‐10 activity significantly decreased the frequency of Ki‐67^+^ cells (Figure 2D,E) and increased active caspase‐3^+^ cells in lesion sections (Figure 2F,G), suggesting that a lack of angiogenesis results in increased cell death and decreased cell proliferation in lesions injected with mAbs against mIL‐10. These data suggest that IL‐10 promotes endometriotic lesion development through enhancing angiogenesis during the early stage of endometriosis.

### The IL‐10—IL‐10R pathway enhances the migratory and tube formation ability in HUVECs

**Figure 3:**
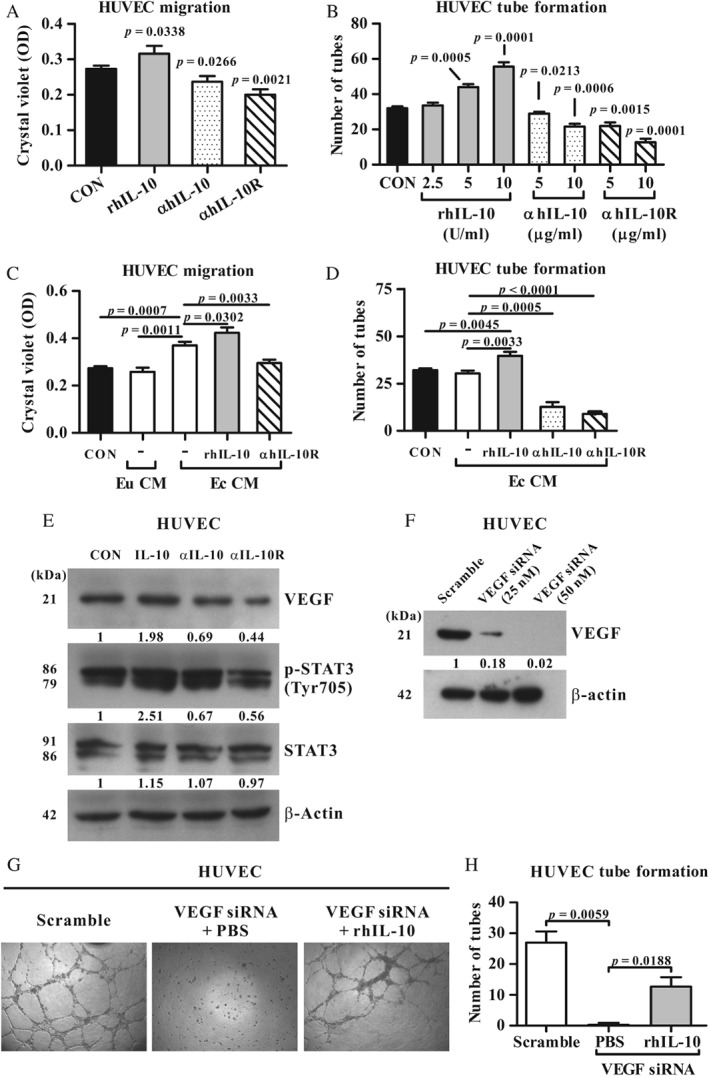
Effect of the IL‐10—IL‐10R pathway on the angiogenic activity of HUVECs *in vitro*. (A, B) HUVECs were treated with medium alone (CON), rhIL‐10 (10 U/ml, unless otherwise indicated), hIL‐10 mAb (10 µg/ml, unless otherwise indicated), or hIL‐10R mAb (10 µg/ml, unless otherwise indicated) for migration assays and tube formation assays. (C, D) CM from treated ectopic EN‐MSCs (Ec) was cultured with HUVECs for the migration assay and the tube formation assay. CM from human eutopic EN‐MSCs (Eu) was used as a cell type control. HUVECs treated with medium alone served as a negative control (CON). (E) Representative western blotting for VEGF, p‐STAT3, total STAT3, and β‐actin in treated HUVEC cells as shown in A. (F) HUVECs were transfected with scrambled siRNA (scramble) or with VEGF‐specific siRNA at 25 or 50 nm for 12 h, followed by western blotting for VEGF and β‐actin in treated HUVECs. Each immunoblot was quantified for the target protein relative to its corresponding β‐actin levels. Results are shown as ratios that were normalised to the corresponding CON sample. These are representative blots from three independent experiments. (G, H) Tube formation of HUVECs after treatment with scrambled siRNA, VEGF siRNA at 25 nm in PBS, or VEGF siRNA at 25 nm together with rhIL‐10 for 12 h. Results (mean ± SD) shown in A–D and H are from three independent experiments.

As it has been reported that endothelial cells can express IL‐10R 31, we next determined whether IL‐10 can directly act on vessel endothelial cells through IL‐10R to stimulate angiogenesis by using conventional HUVEC migration and tube formation assays performed *in vitro*. Transwell experiments showed that the migration of HUVECs was significantly enhanced by rhIL‐10 and significantly decreased by the addition of blocking mAbs against either hIL‐10 or hIL‐10R, compared with the control (Figure 3A and supplementary material, Figure S2A). IL‐10 treatment also significantly increased the number of tubes formed by HUVECs in a dose‐dependent manner; however, the tube formation of HUVECs was inhibited by mAbs against either hIL‐10 or hIL‐10R (Figure 3B and supplementary material, Figure S2B). These results suggest that the IL‐10–IL‐10R pathway stimulates the angiogenic activity of endothelial cells *in vitro*.

### IL‐10 stimulates the migratory ability of ectopic EN‐MSCs

Growing evidence indicates that stem cells are involved in the root aetiology of endometriosis 32. In our previous work, we isolated eutopic and ectopic EN‐MSCs from individual patients with ovarian endometriosis and verified that the unique migration, invasion, and angiogenesis characteristics of ectopic EN‐MSCs may underlie the pathogenesis of ectopic endometriosis 23, 33. Here, we further investigated whether IL‐10 also has the ability to affect the migration ability of human EN‐MSCs. After 24‐h treatment, the migration of ectopic EN‐MSCs was significantly enhanced by rhIL‐10 but was significantly decreased by mAbs against hIL‐10 or hIL‐10R, compared with the control (supplementary material, Figure S3). These results suggest that the IL‐10–IL‐10R pathway directly contributes to the mobility of human EN‐MSCs.

### The IL‐10—IL‐10R pathway can promote HUVEC angiogenesis through soluble factors secreted by ectopic EN‐MSCs

Because IL‐10 can directly enhance the migratory ability of ectopic EN‐MSCs (supplementary material, Figure S3), we next tested whether IL‐10‐treated EN‐MSCs can promote angiogenesis in a paracrine fashion. After rhIL‐10 or blocking mAb treatment, eutopic or ectopic EN‐MSCs were washed and cultured with fresh medium, which was subsequently collected as a source of secreted soluble mediators. The resulting culture media were then incubated with HUVEC cultures for 24 h. As shown in Figure 3C and supplementary material, Figure S4A, compared with CM from eutopic EN‐MSCs, CM from ectopic EN‐MSCs promoted HUVEC migration. In addition, CM from rhIL‐10‐treated ectopic EN‐MSCs significantly enhanced HUVEC migration as well as tube formation, but CM from ectopic EN‐MSCs treated with hIL‐10 or hIL‐10R mAbs significantly inhibited HUVEC migration and tube formation (Figure 3C,D and supplementary material, Figure S4). These data suggest that in response to IL‐10–IL‐10R signalling, ectopic EN‐MSCs stimulate HUVEC angiogenesis in a paracrine fashion.

### VEGF is one of the angiogenic factors released by IL‐10‐stimulated cells

As VEGF is a classic angiogenic factor and its level is significantly elevated in the peritoneal fluid of patients with endometriosis 34, we next examined whether angiogenesis mediated by the IL‐10–IL‐10R pathway was dependent on VEGF secretion from HUVECs or EN‐MSCs. Western blot analysis showed that the VEGF level was modestly increased or obviously decreased after HUVECs were treated with rhIL‐10 or blocking mAbs against hIL‐10 and hIL‐10R, respectively (Figure 3E). As IL‐10 binds to IL‐10R and exerts downstream effects through STAT3, we also observed that rhIL‐10 treatment increased the level of phosphorylated STAT3, whereas blocking hIL‐10 or hIL‐10R decreased its phosphorylation (Figure 3E). Similar to HUVECs, ectopic EN‐MSCs secreted more VEGF in response to rhIL‐10 stimulation and secreted less VEGF in response to treatment with mAbs against hIL‐10 or hIL‐10R, compared with control (supplementary material, Figure S5). In addition, VEGF knockdown experiments showed that HUVEC tube formation was significantly inhibited by a reduction in VEGF expression but profoundly increased when the decrease in VEGF was accompanied by rhIL‐10 treatment (Figure 3F,H). These results demonstrated that the IL‐10–IL‐10R pathway promoted angiogenesis through a VEGF‐dependent pathway(s) as well as an unidentified VEGF‐independent pathway(s) *in vitro*.

### IL‐10 can promote angiogenesis *in vivo*

**Figure 4:**
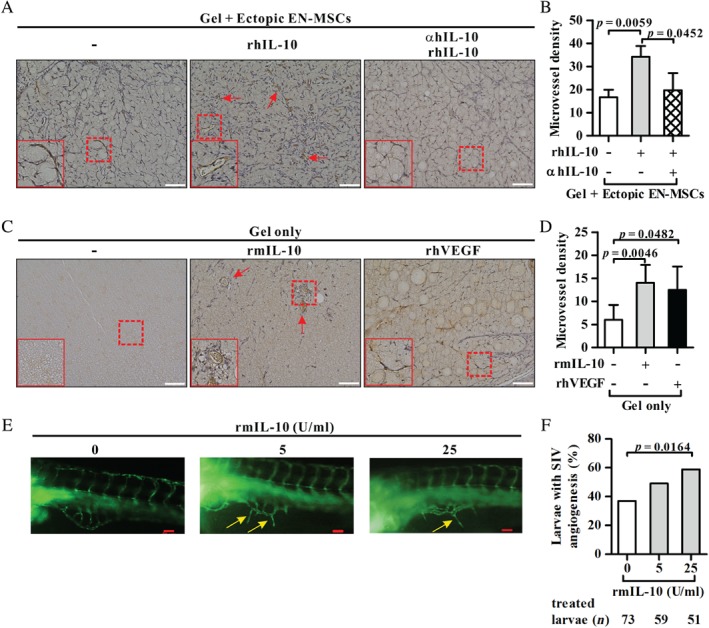
Effect of the IL‐10—IL‐10R pathway on angiogenesis *in vivo*. (A, C) Matrigel plugs containing ectopic EN‐MSCs mixed with recombinant proteins as indicated (A) or recombinant proteins alone as indicated (C) were implanted into female NUDE mice. Representative photomicrographs of paraformaldehyde‐fixed sections from the Matrigel plugs stained with anti‐mCD31 (brown) and haematoxylin (blue) are shown. The insets (solid box) show two‐fold enlarged images of microvascular structures (dashed box). Arrows indicate microvascular structures. Scale bars = 100 µm. (B, D) Microvessel density was evaluated based on the number of CD31^+^ haematoxylin^+^ cells per square millimetre. The experiments were repeated at least three times, and the data are presented as the mean ± SD (*n* = 3 mice in each group in B; *n* = 3–6 mice in each group in D). (E) The yolk sac of 72 h post‐fertilisation zebrafish embryos was injected with PBS or with 5 or 25 U/ml rmIL‐10 in PBS. Representative photographs taken 24 h after injection using an epifluorescence microscope show the angiogenesis of the SIV (yellow arrows) in the resulting zebrafish larvae. Scale bars = 50 µm. The number of treated larvae in each group is indicated. (F) The percentage of larvae with angiogenesis of the SIV is shown for each of the three groups. Student's unpaired *t*‐test was performed in B and D, and Fisher's exact test in F.

We further evaluated the angiogenic activity of IL‐10 *in vivo* using a conventional Matrigel plug assay and a zebrafish model. We implanted into mice Matrigel plugs that contained human ectopic EN‐MSCs mixed with either rhIL‐10 alone or rhIL‐10 that had been pre‐incubated with hIL‐10 mAb. The immunohistochemistry analysis of the Matrigel sections using anti‐mCD31 showed that ectopic EN‐MSCs mixed with rhIL‐10 significantly enhanced microvessel density compared with the cells alone; however, cells mixed with blocked rhIL‐10 inhibited the effect of rhIL‐10 on microvessel density (supplementary material, Figure S6A and Figure 4A,B). Interestingly, in the absence of EN‐MSCs, Matrigel with rmIL‐10 directly promoted an increase in microvessel density compared with buffer (PBS) alone. The elevated microvessel density mediated by rmIL‐10 was similar to that by rhVEGF treatment in the Matrigel plug assay (supplementary material, Figure S6B and Figure 4D). The findings from the *in vivo* assay are consistent with those observed *in vitro* and suggest that the IL‐10–IL‐10R pathway may enhance angiogenesis by acting on at least two cell types, endometriotic cells and vessel endothelial cells, in the context of endometriosis.

A zebrafish model was also used to demonstrate the conserved role of IL‐10 on angiogenesis, as the blood vessel pattern is well characterised in developing zebrafish embryos 35. Microangiography of the subintestinal vessel (SIV) in zebrafish larvae showed that rmIL‐10 significantly increased the percentage of zebrafish larvae with SIV development in a dose‐dependent manner (Figure 4E,F). Taken together, we showed that IL‐10 could promote angiogenesis development in at least two animal models, suggesting its conserved and crucial role in angiogenesis.

### Increased CD123^+^ pDCs, CD31 marker, and IL‐10R expression are observed in human ovarian endometrioma lesions

**Figure 5:**
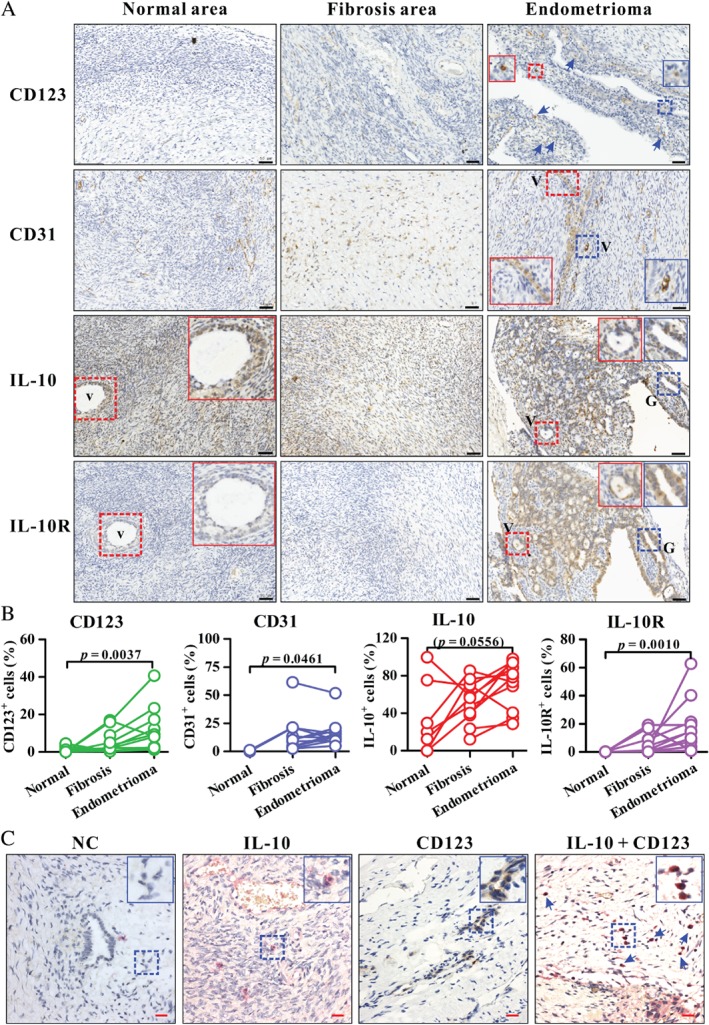
Analysis of CD123, CD31, IL‐10, and IL‐10R expression in human endometrioma and uterine endometrial tissues. (A) Immunohistochemistry for CD123, CD31, IL‐10, or IL‐10R (brown colour) in a representative endometrioma lesion as well as the fibrosis area and normal area surrounding the corresponding endometrioma lesion. The insets (solid box) show two‐fold enlarged images of vascular structure (V) or glandular epithelium (G) (dashed box) with the same colour. Arrows indicate CD123^+^ cells. Blue, haematoxylin counterstain. Black scale bars = 50 µm. The enlarged images of IL‐10 and IL‐10R expression on endometrioma lesions in A are shown in supplementary material, Figure S7 for clear presentation. (B) Quantification of positive cell frequencies in each endometrioma lesion and corresponding fibrosis area and/or normal tissue analysed by HistoQuest software. Number of endometrioma lesion samples: 11 for all markers; number of fibrosis areas within the endometrioma lesion samples: 11 for all markers; number of normal regions within the endometrioma lesion samples: 8 for CD123, 2 for CD31, 7 for IL‐10, and 5 for IL‐10R. *p* < 0.05 was considered significant based on the Kruskal–Wallis test and then Dunn's multiple comparison test. (C) Double immunohistochemical staining for IL‐10 (red) or/and CD123 (brown) in a representative human endometrioma lesion. NC, negative control with omission of both primary antibodies. The insets (solid box) show two‐fold enlarged images of positive cells as indicated (dashed box). Arrows indicate IL‐10^+^ CD123^+^ pDCs with voluminous cytoplasm and smooth cell surface. Blue, haematoxylin counterstain. Red scale bars = 20 µm.

We further explored the infiltration of pDCs, the expression of IL‐10/IL‐10R, and the density of CD31^+^ blood vessel cells in human endometrioma lesions from patients with stage III or IV endometriosis. Compared with the normal tissue surrounding endometrioma lesions, the frequency of pDC (CD123 as a representative marker 36) infiltration, of CD31^+^ cells, and of IL‐10R^+^ cells was significantly increased in the lesions (Figure 5A,B). In an endometriotic lesion with high IL‐10R expression, we found that IL‐10R was expressed both in blood vessels and in glandular epithelium (supplementary material, Figure S7A), but almost no IL‐10R expression was noted in the normal area near the endometrioma lesions (Figure 5A) or in normal uterine endometrium (supplementary material, Figure S8).

With respect to IL‐10 expression in lesions, although it did not reach statistical significance, IL‐10 expression in five of seven matched samples was obviously increased in endometrioma lesions compared with the surrounding normal tissue (Figure 5B). In a representative late‐stage endometriotic lesion, IL‐10 expression was found in multiple cell types, including glandular epithelium, stromal cells, and some endothelial cells of blood vessels (supplementary material, Figure S7B). To identify IL‐10‐secreting pDCs in endometriotic lesions, we performed double immunohistochemical staining for CD123 and IL‐10 molecules. As shown in Figure 5C, we observed IL‐10^+^ CD123^+^ cells with typical pDC morphology, which consists of a slightly more voluminous cytoplasm and a smooth cell surface without dendritic processes 37. Taken together, in support of the *in vitro* and *in vivo* results, human pathology data suggest that the effect of the IL‐10–IL‐10R pathway on angiogenesis is associated with endometrioma development in patients.

## Discussion

Because of the highly complex and dynamic microenvironment, it is still unclear whether immune dysregulation is a cause or a result of endometriosis 16. In the present study, altering the local IL‐10 activity at the beginning of uterine endometrium tissue attachment significantly affected lesion growth and angiogenesis 4 weeks after surgery. Furthermore, IL‐10–IL‐10R signalling enhanced angiogenic activity *in vitro*, based on well‐characterised assays for HUVEC function as well as an *in vivo* Matrigel plug assay and zebrafish model. These results suggest that local high IL‐10 activity may promote lesion implantation and invasion by stimulating angiogenesis as well as suppressing immune surveillance against endometrial debris. In addition, the present study demonstrates that infiltrated pDCs are, at least in part, the cellular source of IL‐10 in the endometriotic milieu.

**Figure 6:**
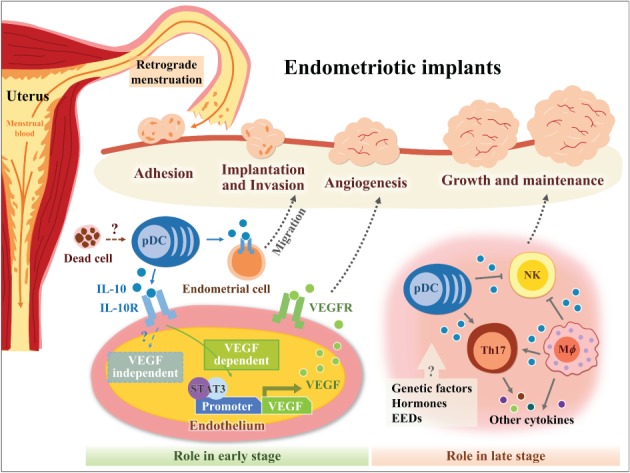
Schematic representation of the effect and immune cell sources of IL‐10 during the early and late stages of endometriosis. During the early implantation and invasion stages, lesion pDCs may secrete IL‐10 in response to unwanted and apoptotic cells. Local IL‐10 further promotes lesion growth by either suppressing anti‐ectopic fragment immunity or stimulating angiogenesis in VEGF‐dependent and ‐independent pathways. In addition, IL‐10—IL‐10R signalling may stimulate endometrial cell migration. The complex interaction among genetic factors, endogenous hormones, environmental endocrine disruptors (EEDs), and impaired immune surveillance leads to chronic inflammation. In the context of chronic inflammation in the peritoneal cavity, other immune cells, such as alternative activated macrophages and Th17 cells as well as endometrial cells (not shown here), can secrete IL‐10 and other mediators to further promote the growth and maintenance of ectopic implants.

Although pDCs may promote tumour development through indoleamine 2,3‐dioxygenase expression 21 and induction of regulatory T‐cell expansion 22, a few studies have reported that pDCs can secrete IL‐10 in response to environmental stimuli. Using IL‐10‐GFP reporter mice for identifying IL‐10‐secreting cells *in vivo*, we recently showed that pDCs are the major immune cell type that expresses IL‐10 within endometriotic lesions in a surgical model 19. The data shown in Figure 1B are consistent with one previous study that showed that TLR7‐activated pDCs can directly respond to apoptotic cells by secreting IL‐10, suggesting that pDCs can maintain self‐tolerance to dying cells within an inflammatory microenvironment 38. In addition, multi‐parametric analysis using flow cytometry showed that the frequency of infiltrated pDCs was significantly higher than that of other immune cell subsets with IL‐10‐secreting ability, such as conventional DCs (cDCs), macrophages, NK cells, and lymphocytes in the endometriotic lesions (supplementary material, Figure S1). We thus speculate that an immune imbalance occurs between eliminating unwanted cells/tissue and maintaining self‐tolerance within the peritoneal cavity in women during the menstrual cycle (Figure 6, ‘Role in early stage’). This immune imbalance may, at least in part, be due to the high expression of IL‐10 in innate cells, such as pDCs, or to the high frequency of infiltrated innate cells during the early establishment of endometriotic lesions. The role of IL‐10‐secreting pDCs in the pathogenesis of endometriosis awaits further investigation.

In addition to pDCs, non‐immune cells seem to express IL‐10 in endometriotic lesions; however, whether IL‐10‐expressing non‐immune cells contribute to the pathogenesis of endometriosis awaits further investigation. Uterine IL‐10 actively suppresses maternal immunity to accept the foetus within the context of pregnancy 39. In normal endometrium in the uterus, both stromal cells and epithelial cells can express *IL10* mRNA irrespective of the phase of the menstrual cycle; however, the level of IL‐10 protein begins to increase significantly in women during early pregnancy 40. In support of the absence of or minimal IL‐10 release in normal endometrium before pregnancy, an IL‐10 immunohistochemical analysis in the normal endometrium sampled from patients with endometrioma showed strong nuclear, but not cytoplasmic, immunoreactivity in glandular and stromal regions (supplementary material, Figure S8A), suggesting that *IL10* expression is tightly regulated in normal endometrium and during pregnancy. Under this tight regulation, it is unclear whether shed endometrium can secrete IL‐10 protein or not in the early stage of endometriosis. In contrast, IL‐10R was barely expressed in normal endometrium and in the normal area surrounding the endometrioma but was significantly expressed in human endometriotic lesions, particularly in the endothelium of blood vessels. This suggests that secreted uterine IL‐10 may act on infiltrated IL‐10R^+^ immune cells to protect foetal allografts in an environment with little or no IL‐10R expression, whereas IL‐10 exerts dual activity in suppressing immunity against unwanted endometrial cells and promoting angiogenesis in the ectopic endometriotic milieu that is rich in IL‐10R.

In addition to the potential role of IL‐10 during the early development of endometriosis provided in the present study, IL‐10 may also substantially contribute to the growth and maintenance of ectopic implants during the late stage of endometriosis, as IL‐10 levels in peritoneal fluid are significantly elevated in patients with stage III or IV endometriosis 41. IL‐10‐expressing immune cell types in peritoneal fluid include alternatively activated macrophages 42, Th17 cells 41, and regulatory T cells 43. Also, in the present study, human pathology data showed that various non‐immune cell types may also express IL‐10 in late‐stage endometriotic lesions, including the glandular epithelium, stromal cells, and some endothelial cells of blood vessels (supplementary material, Figure S7). However, the role and action of IL‐10 on angiogenesis during the late stage of endometriosis are unclear (Figure 6, ‘Role in late stage’).

Tumour microenvironment studies have advanced our knowledge of angiogenesis in recent decades, with a particular focus on the cross‐talk between macrophages and tumour cells 44. The hypoxic tumour microenvironment gives rise to the alternative activation of infiltrated macrophages, originally referred to as M2 macrophages. These M2 macrophages, which are conditioned from tumour‐derived IL‐10 or lactic acid, can secrete pro‐angiogenic mediators to promote angiogenesis, including basic fibroblast growth factor, placental growth factor, PDGF‐BB, and MMP9, as well as VEGF 45, 46, 47. The local IL‐10 from tumour cells or macrophages maintains immune suppression 13, and VEGF produced by macrophages 47 promotes angiogenesis in the microenvironment. Consistent with those tumour studies, the IL‐10R–STAT3 axis drives senescent macrophages to alternative polarisation and consequently promotes angiogenesis in age‐related blinding eye disease 14. In the present study, we further demonstrated that the IL‐10–IL‐10R pathway also has the ability to directly stimulate endothelial cell migration and tube formation, and to enhance VEGF secretion from endothelial cells (Figure 3). Interestingly, VEGF knockdown experiments showed that IL‐10 can rescue HUVEC tube formation when HUVEC‐secreted VEGF is at a very low level (Figure 3G,H), suggesting the existence of a VEGF‐independent mechanism. Based on these findings, it is important to clarify whether and how IL‐10‐mediated angiogenesis, as well as immune suppression, contributes to the development of late‐stage endometriosis. From another perspective, a more detailed understanding of the pro‐angiogenic activity and underlying mechanism of IL‐10 within the context of tumour development or any disease in parallel with angiogenesis and chronic inflammation also awaits further investigation.

There are two limitations in the present study. The first one is that we could not provide evidence showing the role of IL‐10‐expressing pDCs during the adherence stage using the surgery‐induced endometriosis model, as the endometrial tissue was forcedly sutured into the peritoneal cavity in the mouse. The other limitation is that the human endometriotic tissues were all from patients with late‐stage endometriosis. As previously discussed, various IL‐10‐expressing immune cell types and non‐immune cell types have been observed in endometriotic tissues during this late stage; therefore, additional investigations are needed to clarify the role of IL‐10‐pDCs during the early stage of endometriosis in humans.

## Author contributions statement

JLS, EMT, and TCH conceived and designed experiments. YC, YSS, CYH, PS, CCC, and YJC carried out experiments and analysed data. JLS and TCH wrote the draft. JLS, EMT, and TCH revised the paper. All the authors were involved in writing the paper and had final approval of the submitted version.

## Supporting information


**Supplementary materials and methods**
**Figure S1.** Analysis of infiltrated immune subsets in lesions in a surgically induced model
**Figure S2.** Effect of IL‐10‐IL‐10R signalling on the migration and tube formation of HUVECs
**Figure S3.** Effect of IL‐10‐IL‐10R signalling on the migration of human ectopic EN‐MSCs
**Figure S4.** Effect of soluble factors secreted by IL‐10‐treated ectopic EN‐MSCs on HUVEC angiogenesis
**Figure S5.** Effect of the IL‐10‐IL‐10R pathway on VEGF production by HUVECs
**Figure S6.** Effect of recombinant IL‐10 on angiogenesis in Matrigel plug assays
**Figure S7.** The IL‐10R or IL‐10‐expressing non‐immune cell types in human endometrioma
**Figure S8.** The expression of IL‐10 or IL‐10R in normal uterine endometrium and human endometrioma tissues
**Table S1.** Demographic characteristics of the patients with endometriosis

## References

[1] Giudice LC , Kao LC . Endometriosis. Lancet 2004; 364: 1789–1799. 15541453 10.1016/S0140-6736(04)17403-5

[2] Bulun SE . Endometriosis. N Engl J Med 2009; 360: 268–279. 19144942 10.1056/NEJMra0804690

[3] Sasson IE , Taylor HS . Stem cells and the pathogenesis of endometriosis. Ann N Y Acad Sci 2008; 1127: 106–115. 18443337 10.1196/annals.1434.014 PMC3107843

[4] Sampson JA . Metastatic or embolic endometriosis, due to the menstrual dissemination of endometrial tissue into the venous circulation. Am J Pathol 1927; 3: 93–110. 19969738 PMC1931779

[5] Hadfield RM , Mardon HJ , Barlow DH , *et al* Endometriosis in monozygotic twins. Fertil Steril 1997; 68: 941–942. 9389831 10.1016/s0015-0282(97)00359-2

[6] Simpson JL , Bischoff FZ . Heritability and molecular genetic studies of endometriosis. Ann N Y Acad Sci 2002; 955: 239–251 discussion 293–235, 396–406. 11949952 10.1111/j.1749-6632.2002.tb02785.x

[7] D'Hooghe TM . Clinical relevance of the baboon as a model for the study of endometriosis. Fertil Steril 1997; 68: 613–625. 9341599 10.1016/s0015-0282(97)00277-x

[8] Lebovic DI , Mueller MD , Taylor RN . Immunobiology of endometriosis. Fertil Steril 2001; 75: 1–10. 11163805 10.1016/s0015-0282(00)01630-7

[9] Gazvani R , Templeton A . Peritoneal environment, cytokines and angiogenesis in the pathophysiology of endometriosis. Reproduction 2002; 123: 217–226. 11866688 10.1530/rep.0.1230217

[10] Li MO , Flavell RA . Contextual regulation of inflammation: a duet by transforming growth factor‐beta and interleukin‐10. Immunity 2008; 28: 468–476. 18400189 10.1016/j.immuni.2008.03.003

[11] Madan R , Demircik F , Surianarayanan S , *et al* Nonredundant roles for B cell‐derived IL‐10 in immune counter‐regulation. J Immunol 2009; 183: 2312–2320. 19620304 10.4049/jimmunol.0900185 PMC2772089

[12] Boonstra A , Rajsbaum R , Holman M , *et al* Macrophages and myeloid dendritic cells, but not plasmacytoid dendritic cells, produce IL‐10 in response to MyD88‐ and TRIF‐dependent TLR signals, and TLR‐independent signals. J Immunol 2006; 177: 7551–7558. 17114424 10.4049/jimmunol.177.11.7551

[13] Mittal SK , Roche PA . Suppression of antigen presentation by IL‐10. Curr Opi Immunol 2015; 34: 22–27. 10.1016/j.coi.2014.12.009 PMC4444374 25597442

[14] Nakamura R , Sene A , Santeford A , *et al* IL10‐driven STAT3 signalling in senescent macrophages promotes pathological eye angiogenesis. Nat Commun 2015; 6: 7847. 26260587 10.1038/ncomms8847 PMC4918330

[15] Dace DS , Khan AA , Kelly J , *et al* Interleukin‐10 promotes pathological angiogenesis by regulating macrophage response to hypoxia during development. PLoS One 2008; 3: e3381. 18852882 10.1371/journal.pone.0003381 PMC2557127

[16] Symons LK , Miller JE , Kay VR , *et al* The immunopathophysiology of endometriosis. Trends Mol Med 2018; 24: 748–762. 30054239 10.1016/j.molmed.2018.07.004

[17] Adams RH , Alitalo K . Molecular regulation of angiogenesis and lymphangiogenesis. Nat Rev Mol Cell Biol 2007; 8: 464–478. 17522591 10.1038/nrm2183

[18] Ahn SH , Monsanto SP , Miller C , *et al* Pathophysiology and immune dysfunction in endometriosis. BioMed Res Internat 2015; 2015: 795976. 10.1155/2015/795976 PMC4515278 26247027

[19] Suen JL , Chang Y , Chiu PR , *et al* Serum level of IL‐10 is increased in patients with endometriosis, and IL‐10 promotes the growth of lesions in a murine model. Am J Pathol 2014; 184: 464–471. 24326257 10.1016/j.ajpath.2013.10.023

[20] Diebold SS , Kaisho T , Hemmi H , *et al* Innate antiviral responses by means of TLR7‐mediated recognition of single‐stranded RNA. Science 2004; 303: 1529–1531. 14976261 10.1126/science.1093616

[21] Munn DH , Sharma MD , Hou D , *et al* Expression of indoleamine 2,3‐dioxygenase by plasmacytoid dendritic cells in tumor‐draining lymph nodes. J Clin Invest 2004; 114: 280–290. 15254595 10.1172/JCI21583 PMC449750

[22] Faget J , Bendriss‐Vermare N , Gobert M , *et al* ICOS‐ligand expression on plasmacytoid dendritic cells supports breast cancer progression by promoting the accumulation of immunosuppressive CD4^+^ T cells. Cancer Res 2012; 72: 6130–6141. 23026134 10.1158/0008-5472.CAN-12-2409

[23] Kao AP , Wang KH , Chang CC , *et al* Comparative study of human eutopic and ectopic endometrial mesenchymal stem cells and the development of an *in vivo* endometriotic invasion model. Fertil Steril 2011; 95: 1308–1315.e1301. 21047634 10.1016/j.fertnstert.2010.09.064

[24] Staton CA , Reed MW , Brown NJ . A critical analysis of current *in vitro* and *in vivo* angiogenesis assays. Int J Exp Pathol 2009; 90: 195–221. 19563606 10.1111/j.1365-2613.2008.00633.x PMC2697546

[25] Akhtar N , Dickerson EB , Auerbach R . The sponge/Matrigel angiogenesis assay. Angiogenesis 2002; 5: 75–80. 12549862 10.1023/a:1021507031486

[26] Revised American Society for Reproductive Medicine classification of endometriosis: 1996. Fertil Steril 1997; 67: 817–821. 9130884 10.1016/s0015-0282(97)81391-x

[27] Karlsson J , von Hofsten J , Olsson PE . Generating transparent zebrafish: a refined method to improve detection of gene expression during embryonic development. Mar Biotechnol (NY) 2001; 3: 522–527. 14961324 10.1007/s1012601-0053-4

[28] Edwards AK , Nakamura DS , Virani S , *et al* Animal models for anti‐angiogenic therapy in endometriosis. J Reprod Immunol 2013; 97: 85–94. 23432875 10.1016/j.jri.2012.10.012

[29] Kitazume S , Imamaki R , Ogawa K , *et al* Sweet role of platelet endothelial cell adhesion molecule in understanding angiogenesis. Glycobiology 2014; 24: 1260–1264. 25214153 10.1093/glycob/cwu094

[30] Vermeulen PB , Gasparini G , Fox SB , *et al* Quantification of angiogenesis in solid human tumours: an international consensus on the methodology and criteria of evaluation. Eur J Cancer 1996; 32A: 2474–2484. 9059336 10.1016/s0959-8049(96)00379-6

[31] Gleissner CA , Zastrow A , Klingenberg R , *et al* IL‐10 inhibits endothelium‐dependent T cell costimulation by up‐regulation of ILT3/4 in human vascular endothelial cells. Eur J Immunol 2007; 37: 177–192. 17163451 10.1002/eji.200636498

[32] Hufnagel D , Li F , Cosar E , *et al* The role of stem cells in the etiology and pathophysiology of endometriosis. Semin Reprod Med 2015; 33: 333–340. 26375413 10.1055/s-0035-1564609 PMC4986990

[33] Kao AP , Wang KH , Long CY , *et al* Interleukin‐1β induces cyclooxygenase‐2 expression and promotes the invasive ability of human mesenchymal stem cells derived from ovarian endometrioma. Fertil Steril 2011; 96: 678–684 e671. 21762900 10.1016/j.fertnstert.2011.06.041

[34] McLaren J , Prentice A , Charnock‐Jones DS , *et al* Vascular endothelial growth factor (VEGF) concentrations are elevated in peritoneal fluid of women with endometriosis. Hum Reprod 1996; 11: 220–223. 8671190 10.1093/oxfordjournals.humrep.a019023

[35] Serbedzija GN , Flynn E , Willett CE . Zebrafish angiogenesis: a new model for drug screening. Angiogenesis 1999; 3: 353–359. 14517415 10.1023/a:1026598300052

[36] Lombardi VC , Khaiboullina SF . Plasmacytoid dendritic cells of the gut: relevance to immunity and pathology. Clin Immunol 2014; 153: 165–177. 24769378 10.1016/j.clim.2014.04.007 PMC4063559

[37] Marafioti T , Paterson JC , Ballabio E , *et al* Novel markers of normal and neoplastic human plasmacytoid dendritic cells. Blood 2008; 111: 3778–3792. 18218851 10.1182/blood-2007-10-117531

[38] Simpson J , Miles K , Trub M , *et al* Plasmacytoid dendritic cells respond directly to apoptotic cells by secreting immune regulatory IL‐10 or IFN‐α. Front Immunol 2016; 7: 590. 28018356 10.3389/fimmu.2016.00590 PMC5155015

[39] Thaxton JE , Sharma S . Interleukin‐10: a multi‐faceted agent of pregnancy. Am J Reprod Immunol 2010; 63: 482–491. 20163400 10.1111/j.1600-0897.2010.00810.x PMC3628686

[40] Vigano P , Somigliana E , Mangioni S , *et al* Expression of interleukin‐10 and its receptor is up‐regulated in early pregnant versus cycling human endometrium. J Clin Endocrinol Metab 2002; 87: 5730–5736. 12466379 10.1210/jc.2002-020435

[41] Chang KK , Liu LB , Jin LP , *et al* IL‐27 triggers IL‐10 production in Th17 cells via a c‐Maf/RORγt/Blimp‐1 signal to promote the progression of endometriosis. Cell Death Dis 2017; 8: e2666. 28300844 10.1038/cddis.2017.95 PMC5386585

[42] Bacci M , Capobianco A , Monno A , *et al* Macrophages are alternatively activated in patients with endometriosis and required for growth and vascularization of lesions in a mouse model of disease. Am J Pathol 2009; 175: 547–556. 19574425 10.2353/ajpath.2009.081011 PMC2716955

[43] Li MQ , Wang Y , Chang KK , *et al* CD4^+^Foxp3^+^ regulatory T cell differentiation mediated by endometrial stromal cell‐derived TECK promotes the growth and invasion of endometriotic lesions. Cell Death Dis 2014; 5: e1436. 25275597 10.1038/cddis.2014.414 PMC4649519

[44] Dehne N , Mora J , Namgaladze D , *et al* Cancer cell and macrophage cross‐talk in the tumor microenvironment. Curr Opin Pharmacol 2017; 35: 12–19. 28538141 10.1016/j.coph.2017.04.007

[45] Spiller KL , Anfang RR , Spiller KJ , *et al* The role of macrophage phenotype in vascularization of tissue engineering scaffolds. Biomaterials 2014; 35: 4477–4488. 24589361 10.1016/j.biomaterials.2014.02.012 PMC4000280

[46] Jetten N , Verbruggen S , Gijbels MJ , *et al* Anti‐inflammatory M2, but not pro‐inflammatory M1 macrophages promote angiogenesis *in vivo* . Angiogenesis 2014; 17: 109–118. 24013945 10.1007/s10456-013-9381-6

[47] Colegio OR , Chu NQ , Szabo AL , *et al* Functional polarization of tumour‐associated macrophages by tumour‐derived lactic acid. Nature 2014; 513: 559–563. 25043024 10.1038/nature13490 PMC4301845

