# Supplementary material for: IL‐10 from plasmacytoid dendritic cells promotes angiogenesis in the early stage of endometriosis
Source: J Pathol. 2019 Oct 6;249(4):485–97. doi: 10.1002/path.5339 (PMC6899974; doi:10.1002/path.5339)
Supplement: Supplementary file 1 — Supplementary materials and methods Figure S1. Analysis of infiltrated immune subsets in lesions in a surgically induced model Figure S2. Effect of IL‐10‐IL‐10R signalling on the migration and tube formation of HUVECs Figure S3. Effect of IL‐10‐IL‐10R signalling on the migration of human ectopic EN‐MSCs Figure S4. Effect of soluble factors secreted by IL‐10‐treated ectopic EN‐MSCs on HUVEC angiogenesis Figure S5. Effect of the IL‐10‐IL‐10R pathway on VEGF production by HUVECs Figure S6. Effect of recombinant IL‐10 on angiogenesis in Matrigel plug assays Figure S7. The IL‐10R or IL‐10‐expressing non‐immune cell types in human endometrioma Figure S8. The expression of IL‐10 or IL‐10R in normal uterine endometrium and human endometrioma tissues Table S1. Demographic characteristics of the patients with endometriosis [file PATH-249-485-s001.doc]

**IL-10 from plasmacytoid dendritic cells promotes angiogenesis in the early stage of endometriosis**

Suen J-L *et al. J Pathol* DOI: 10.1002/path5339

**Supplementary materials and methods**

Reference numbers refer to the main text reference list

*Mice and the surgical endometriosis model*

The protocol was approved by and adhered to the regulations of the Institutional Animal Care and Use Committee of the Kaohsiung Medical University (Permit Number: 103065). C57BL/6 mice or NUDE (CAnN.Cg-*Foxn1^nu^*/CrlNarl) mice from the National Laboratory Animal Center (Taipei, Taiwan) and IL-10 knockout mice (*Il10*^−/−^) from Jackson Laboratory (Bar Harbor, ME, USA) were maintained by the Animal Center of Kaohsiung Medical University in a pathogen-free facility. Female mice at the age of 6–8 weeks were used for experiments. The endometriosis model was established according to our previous study [19]. In brief, autologous uterine horns from female mice were punched to generate four identically sized round tissue samples (2 mm in diameter), and two samples were then surgically sutured to each side of the abdominal wall. Recombinant murine IL-10 (rmIL-10; 100 U/ml, 20 μl per lesion) (Pepro Tech, Rocky Hill, NJ, USA) or blocking mAb against murine IL-10 (mIL-10; 20 μg/ml, 20 μl per lesion) (BD Biosciences, Taipei, Taiwan) was injected into the peritoneum under each transplanted tissue on the right side, with tissue on the left side, which was injected with PBS or the isotype control Ab, serving as the control. Four weeks after surgery, the lesions were collected and weighed, and the lesion area was measured using free image analysis software (ImageJ Software).

*pDC purification and treatment*

Splenocytes were negatively selected for pDCs using a commercial kit according to the manufacturer’s protocol (Miltenyi Biotec, Auburn, CA, USA). The phenotype and purity of viable splenic pDCs (90–95%) were analysed by a flow cytometer (LSR II, BD Biosciences). Purified splenic pDCs (CD11c^low^ PDCA-1^+^; 2 × 10^4^ cells) from *Il10*^−/−^ or control littermates were intravenously transferred into C57BL/6 recipient mice 1 day before surgery.

*Co-culture of apoptotic cells and DC subsets*

Thymocytes isolated from 4- to 6-week-old naïve female C57BL/6 mice were treated with dexamethasone (1 × 10^−7^ m) for 6  h to generate apoptotic cells. The level of apoptotic cells was analysed by flow cytometry using Annexin V-FITC and Violet dye (both kits were from Invitrogen, Carlsbad, CA, USA). The percentage of apoptotic thymocytes used in co-culture experiments was at least 25% for early apoptotic cells (Annexin V^+^ Violet^−^) and 15% for late apoptotic cells (Annexin V^+^ Violet^+^). Splenic pDCs were co-cultured with apoptotic cells at a ratio of 1:100 for 72 h in the presence of R848 (100 nm; Santa Cruz Biotechnology, Dallas, TX, USA), a TLR7 and TLR8 agonist. After 72 h of co-culture, the supernatant was harvested for IL-10 analysis using an ELISA kit (R&D Systems, Minneapolis, MN, USA).

*Flow cytometry*

For preparation of single-cell suspensions, the lesions were incubated for 40 min in 0.05% trypsin, 0.53 mm EDTA, 0.1% collagenase D, and 150 mg/ml DNase I, and mechanically disrupted with a gentleMACS Dissociator (Miltenyi Biotec) according to the manufacturer’s instructions. The single-cell suspensions were stained with fluorochrome-labelled mAbs against CD45 (30-F11; BD Biosciences), PDCA-1 (eBio927; eBioscience, San Diego, CA, USA), CD11c (N418; eBioscience), B220 (RA3-6B2; BD Biosciences), CD3 (145-2C11; eBioscience), and NK1.1 (PK136; eBioscience) to characterise DC subsets and for lymphocyte staining. The cells were also stained with fluorochrome-labelled mAbs against F4/80 (BM8; Biolegend, San Diego, CA, USA), Ly6c (HK1.4; Biolegend), CD11b (M1/70; Biolegend), and CD45 (30-F11; eBioscience) for macrophage and monocyte staining. The cellular composition of lesion cells was analysed by flow cytometry (LSR II; BD Biosciences) and FlowJo software (version 10; Tree Star, Inc, Ashland, OR, USA).

*Immunofluorescence of murine lesions*

Serial 3-μm-thick sections from murine frozen endometriotic lesions were fixed with 4% paraformaldehyde, and antigens were retrieved by incubation in sodium citrate buffer for 5 min in boiling water. The slides were incubated with rabbit anti-mouse CD31 (anti-mCD31, 20 μg/ml; Abcam, Cambridge, UK), a specific endothelial cell marker, overnight at 4°C. For control staining, only buffer was added to the tissue sections. The washed slides were then stained with Alexa Fluor 568 goat anti-rabbit IgG (2 μg/ml; Thermo Fisher Scientific, Waltham, MA, USA) for 1 h at room temperature. Cell nuclei were stained with DAPI (1 μg/ml; Sigma-Aldrich, St Louis, MO, USA). The stained lesions were scanned with the TissueFAXS imaging system (TissueGnostics, Tarzana, CA, USA), with which a series of separate images per fluorescence channel and field of view was taken, collected, and merged automatically into a virtual sample. The CD31^+^ DAPI^+^ cells in all fields were analysed with TissueQuest software (TissueGnostics). DAPI (nuclear stain) was defined as the master marker, and Alexa Fluor 568 (CD31) as the non-master marker. The cut-off line was determined by lesions stained with incubation buffer alone (i.e. without the primary antibody, anti-mCD31).

*Immunohistochemistry* *of human endometrioma lesions and quantitative analysis by TissueFAXS*

Immunostaining of endometrioma or uterine endometrial tissues for human CD123 (BR4MS; Leica Biosystems, Wetzlar, Germany), human CD31 (GM006; Genemed Biotechnologies, Inc, Maidenhead, UK), human IL-10 (orb10892; Biorbyt, San Francisco, CA, USA), human IL-10RA (ab197666; Abcam), or murine lesions for mouse Ki-67 (AB9260; Merck KGaA, Darmstadt, Germany), and mouse active caspase-3 (AB3623; Merck KGaA) was performed with the fully automated Bond-Max system (Leica Microsystems). Slides carrying tissue sections from formalin-fixed, paraffin-embedded tissue microarray blocks were dried for 1 h at 60°C. These slides were then covered by Bond Universal Covertiles and placed into the Bond-Max instrument. All subsequent steps were performed by the automated instrument according to the manufacturer’s instructions (Leica Microsystems). In brief, the steps were as follows: (1) deparaffinisation of tissue on the slides by rinsing with Bond Dewax Solution at 72°C; (2) heat-induced epitope retrieval (antigen unmasking) with Bond Epitope Retrieval Solution 1 or 2 for 20 min at 100°C; (3) peroxide block placement on the slides for 5 min at room temperature; (4) incubation with primary antibodies against human CD123, CD31, IL-10, or IL-10R at a dilution of 1:50, 1:200, 1:100, or 1:100, respectively, for 30 min at room temperature; (5) Bond Polymer placement on the slides for 8 min at room temperature; (6) colour development with DAB as the chromogen for 5 min at room temperature; and (7) haematoxylin counterstaining for 5 min, followed by coverslipping of the slides.

For double immunohistochemical staining, samples were dewaxed and rehydrated, and underwent antigen retrieval with EDTA buffer (pH 9) for 30 min at 95°C. The slides were then stained with primary antibody against human CD123 (BR4MS; Leica Biosystems) at a dilution of 1:50 and then stained with Novolink Polymer DS (RE7290-CE; Leica Biosystems). After being washed, the slides were stained with antibody against human IL-10 (ab34843; Abcam) at a dilution of 1:400, followed by staining with UltraTek Alk-Phos (AMH080-IFU; ScyTek, Logan, UT, USA) and then with haematoxylin.

Images of the stained tissue were captured by the TissueFAXS imaging system (TissueGnostics) and were then processed using HistoQuest. Haematoxylin staining was used as a master marker for cell identification on the basis of nuclear detection. Furthermore, the average nuclear size, discrimination area, discrimination grey, and background threshold for the master marker were specified. The range of intensities of the master marker (haematoxylin) and the immunohistochemical staining was set by auto-detection with the software. Regions of interest were manually defined for each area by the pathologist, Dr Yi-Ju Chen, at E-Da Hospital.

*HUVEC cell culture*

HUVECs were obtained from the American Type Culture Collection and maintained in Endothelial Cell Medium (ScienCell Research Laboratories, San Diego, CA, USA) supplemented with 5% FBS (Gibco-Life Technologies, Carlsbad, CA, USA), 1% endothelial cell growth supplement, 1% penicillin/streptomycin solution (ScienCell Research Laboratories), and 1% l-glutamine (Thermo Fisher Scientific) at 37°C in a humidified 5% CO_2_ atmosphere. Cells were used for experiments during passages 3–5.

*Endometrial mesenchymal stem cell (EN-MSC) culture*

The procedure used to isolate EN-MSCs from human uterine eutopic endometrium and ectopic endometriotic lesions has been described previously [23]. In brief, the tissues were minced and digested with type II collagenase (Thermo Fisher Scientific), and then stromal cells were separated from epithelial glands by wire sieves. To isolate EN-MSCs, large colonies of stromal cells were selected and processed via limiting dilution, and colony formation potential was subsequently assessed. The EN-MSCs were characterised using MSC phenotypes: differentiation, induction, and gene expression. EN-MSCs were cultured in a 2:1 (v/v) mixture of Dulbecco’s Modified Eagle’s Medium: Nutrient Mixture F12 (Gibco-Life Technologies) and MCDB 153 medium (Keratinocyte-SFM; Gibco-Life Technologies) supplemented with 10% FBS, 2 mm *N*-acetyl-l-cysteine (Sigma-Aldrich), and 0.2 mm l-ascorbic acid-2-phosphate (Sigma-Aldrich).

*Transwell migration assay*

HUVECs or ectopic EN-MSCs were conditioned overnight in Endothelial Cell Medium (ScienCell Research Laboratories, San Diego, CA, USA). The cells were then seeded at 2 × 10^4^ cells per well into the upper compartment of chambers containing rhIL-10 or blocking mAbs (inserts; 8-μm pore size; Merck KGaA). The bottom chambers were filled with Endothelial Cell Medium supplemented with 5% FBS, 1% endothelial cell growth supplement, 1% penicillin/streptomycin solution, and 1% l-glutamine. After 24-h incubation, the membranes were washed and fixed with 3.7% paraformaldehyde and stained with 0.5% crystal violet. The basal side of each membrane was visualised with a Zeiss Axiovert 200M microscope at 100× magnification. The membranes were washed, and the dye was eluted with 10% acetic acid. The absorbance (OD) was measured at 595 nm using an ELISA reader (Dynex MRX TC, Labequip ABEQUIP).

*Tube formation assay*

A suspension of 20 000 HUVECs or ectopic EN-MSCs was seeded in triplicate into Matrigel-precoated 24-well plates in the presence of rhIL-10 (10 U/ml), hIL-10 mAb (10 μg/ml; eBioscience), or hIL-10R mAb (10 μg/ml; eBioscience) for 12 h. The formation of tubes was evaluated and statistically analysed as described previously [24].

*Transfection of siRNAs and sequences of siRNAs*

Cells (4 × 10^4^) were transfected with 25 nm VEGF siRNA or with non-silencing (medium GC Duplex) negative control siRNA by using Lipofectamine 2000 (Thermo Fisher Scientific) for 6 h. At 48 h after the transfections, these cells were collected and the protein levels of the siRNA targets assessed by immunoblotting. The VEGF-specific siRNAs were predesigned Stealth siRNA oligonucleotides targeting *VEGFA* (HSS111274) (Thermo Fisher Scientific), and the sequences were 5′-UUGCAUCAUAGUUAGAUAAGACUGC-3′ and 5′-GCAGUCUUAUCUAACUAUGAUGCAA-3′.

*Western blotting*

HUVECs were resuspended in RIPA lysis buffer (Thermo Fisher Scientific) and protein concentrations were determined using a Bio-Rad Protein assay (Bio-Rad Laboratories, Hercules, CA, USA). Equal amounts of protein were diluted with 4× loading buffer, incubated in a boiling water bath for 5 min, separated on a 12% SDS-polyacrylamide gel, and transferred to PVDF membranes (Merck KGaA). After blocking with 5% milk, membranes were incubated with primary antibodies overnight at 4°C, followed by HRP-conjugated secondary antibodies (Jackson ImmunoResearch, West Grove, PA, USA) and ECL (Merck KGaA). Primary antibodies against STAT3 and VEGF were purchased from Santa Cruz Biotechnology, p-STAT3 (Tyr705) was from Cell Signaling Technology (Danvers, MA, USA), and β-actin was from GeneTex (Irvine, CA, USA).

In vivo *Matrigel plug assay and histological examination*

Female NUDE (CAnN.Cg-*Foxn1nu*/CrlNarl) mice were subcutaneously injected with 250 μl of an ice-cold Matrigel mixture at a final concentration of 10 mg/ml (BD Biosciences). After 7 days, the mice were euthanised and dissected to remove the Matrigel plugs. Tissue sections from the Matrigel plugs were incubated with anti-mCD31, followed by HRP-conjugated anti-rabbit IgG and DAB (Bio SB, Santa Barbara, CA, USA) according to the manufacturer’s instructions, and were then counterstained with haematoxylin [25]. Microvessel density was evaluated based on the number of CD31^+^ haematoxylin^+^ cells per tissue area (in millimetres squared).

*Zebrafish model*

The protocol was approved by and adhered to the regulations of the Institutional Animal Care and Use Committee of the Kaohsiung Medical University (Permit Number: 107066). To prevent pigment formation and maintain transparency for image observation, 24 hpf (hour post-fertilisation) zebrafish larvae were pretreated with 1-phenyl 2-thiourea [27]. At 72 hpf, a total volume of 5 nl of the indicated dose of rmIL-10 was injected into the yolk sac of individual zebrafish larvae, which were then incubated at 28.5°C for 24 h. PBS served as a control. The effect of IL-10 on angiogenesis of the subintestinal vessel (SIV) in zebrafish larvae was observed using an epifluorescence microscope (TE-2000, Nikon).

**
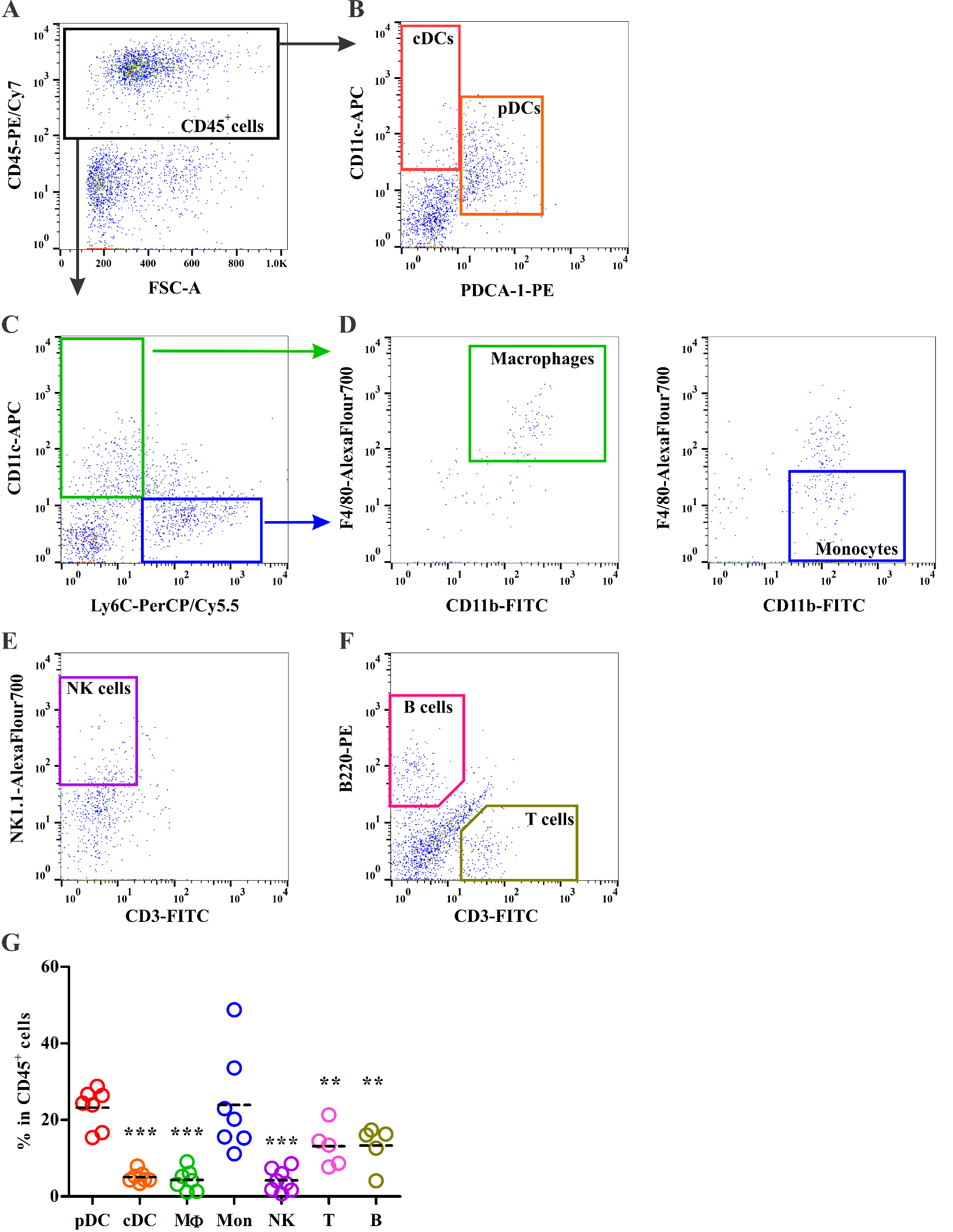
**

**Figure S1.** Analysis of infiltrated immune subsets in lesions in a surgically induced model. (A–F) Lesions were isolated from C57BL/6 mice 4 weeks after surgery, and single cells were analysed using flow cytometry for viable cells. The sequential gating strategy consisted of CD45^+^ cells (A), pDCs (CD11c^+^ PDCA-1^+^) and cDCs (CD11c^+^ PDCA-1^−^) (B), macrophages (CD11c^+^ Ly6C^−^ CD11b^+^ F4/80^high^) and monocytes (CD11c^−^ Ly6C^+^ CD11b^+^ F4/80^low^) (C, D), natural killer (NK) cells (CD3^−^ NK1.1^+^) (E), and T cells (CD3^+^ B220^−^) and B cells (CD3^−^ B220^+^) (F). (G) The frequency of various cell subsets among viable CD45^+^ cells in pooled lesions from each mouse. Mφ, macrophages; Mon, monocytes; NK, NK cells; T, T cells; B, B cells. *n* = 5–8 mice, with all lesions per mouse. ***p* < 0.01, ****p* < 0.001 versus pDC.


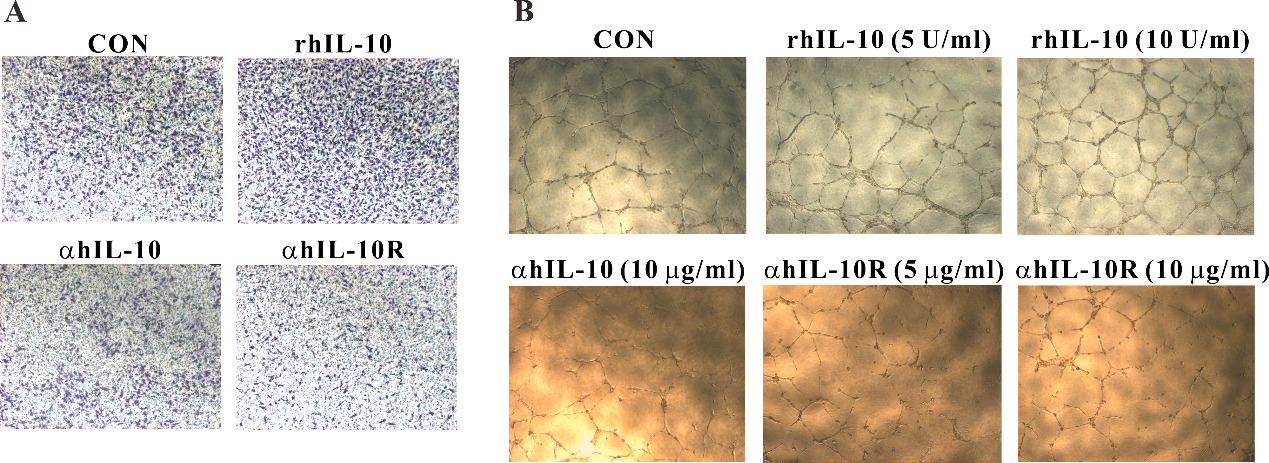


**Figure S2.** Effect of IL-10−IL-10R signalling on the migration and tube formation of HUVECs. (A) HUVECs were treated with medium alone (CON), rhIL-10 (10 U/ml), hIL-10 mAb (10 μg/ml), or hIL-10R mAb (10 μg/ml) for 24 h, and cells that migrated through the inserts were analysed using crystal violet staining. (B) HUVECs were treated as indicated for 12 h and photographed by microscope for quantification of tube numbers. Representative images from three replicates in a single independent experiment are shown. The quantification results of A and B are presented in Figure 3A,B, respectively.

**
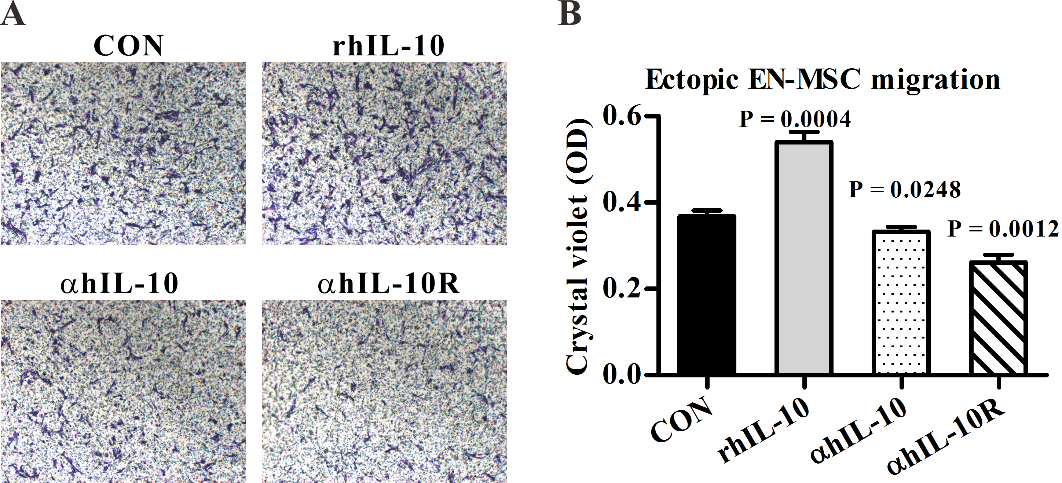
**

**Figure S3.** Effect of IL-10−IL-10R signalling on the migration of human ectopic EN-MSCs. Human ectopic EN-MSCs were treated with medium alone (CON), rhIL-10 (10 U/ml), hIL-10 mAb (10 μg/ml), or hIL-10R mAb (10 μg/ml) for 24 h, and cells that migrated through the inserts were analysed using crystal violet staining. Results are from three independent experiments and are shown as the mean ± SD.

**
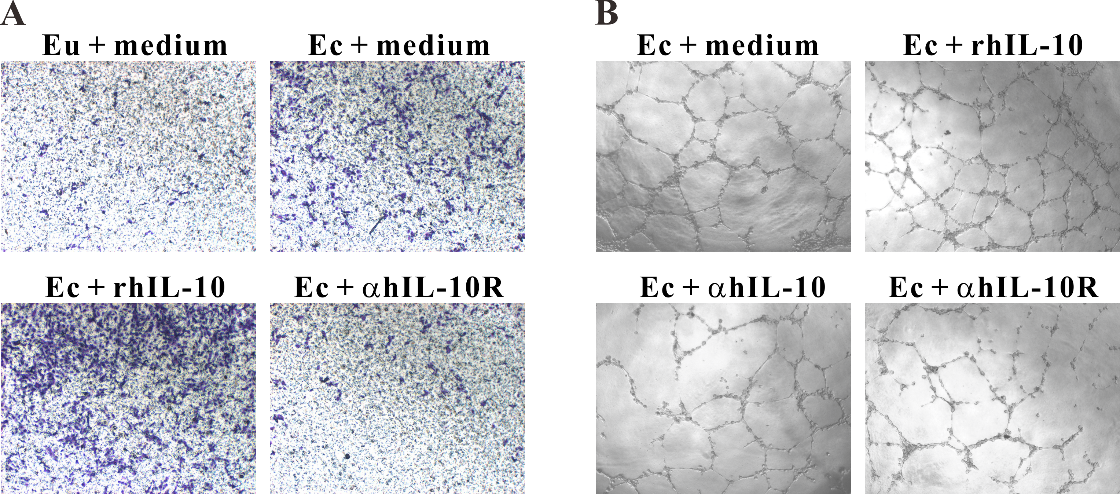
**

**Figure S4.** Effect of soluble factors secreted by IL-10-treated ectopic EN-MSCs on HUVEC angiogenesis. Human ectopic EN-MSCs (Ec) were treated with medium plus rhIL-10 (10 U/ml), hIL-10 mAb (10 μg/ml), or hIL-10R mAb (10 μg/ml) for 24 h, after which all cultures were incubated with fresh medium alone without any treatment for an additional 24 h. The CM was used to culture HUVECs before assessing their migration (A) and tube formation (B). Human eutopic EN-MSCs (Eu) were used as a cell type control. Representative images from three replicates in an independent experiment are shown. The quantification results of A and B are presented in Figure 3C,D, respectively.


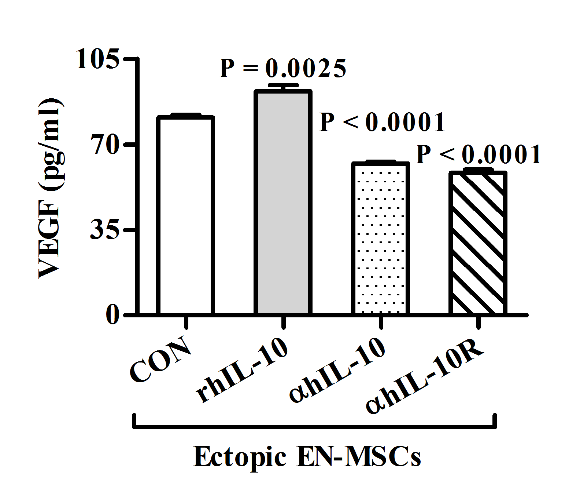


**Figure S5.** Effect of the IL-10−IL-10R pathway on VEGF production by HUVECs. Ectopic EN-MSCs were treated with medium alone (CON), rhIL-10 (10 U/ml), hIL-10 mAb (10 μg/ml), or hIL-10R mAb (10 μg/ml) for 24 h. VEGF levels in the supernatants were analysed by ELISAs. Results are from three independent experiments and are shown as the mean ± SD.


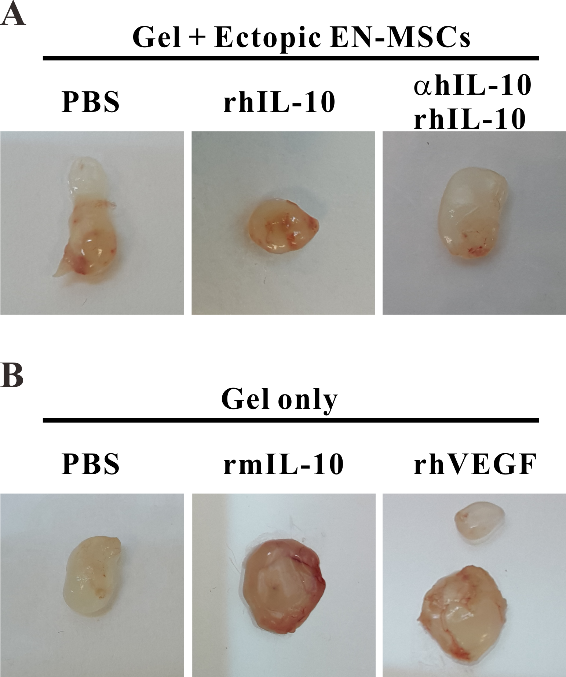


**Figure S6.** Effect of recombinant IL-10 on angiogenesis in Matrigel plug assays. Matrigel plugs containing ectopic EN-MSCs admixed with recombinant proteins as indicated (A) or containing recombinant proteins alone as indicated (B) were implanted into female NUDE mice. Representative plugs from at least three independent experiments are shown. The quantification results (microvessel density) of A and B are presented in Figure 4B,D, respectively.


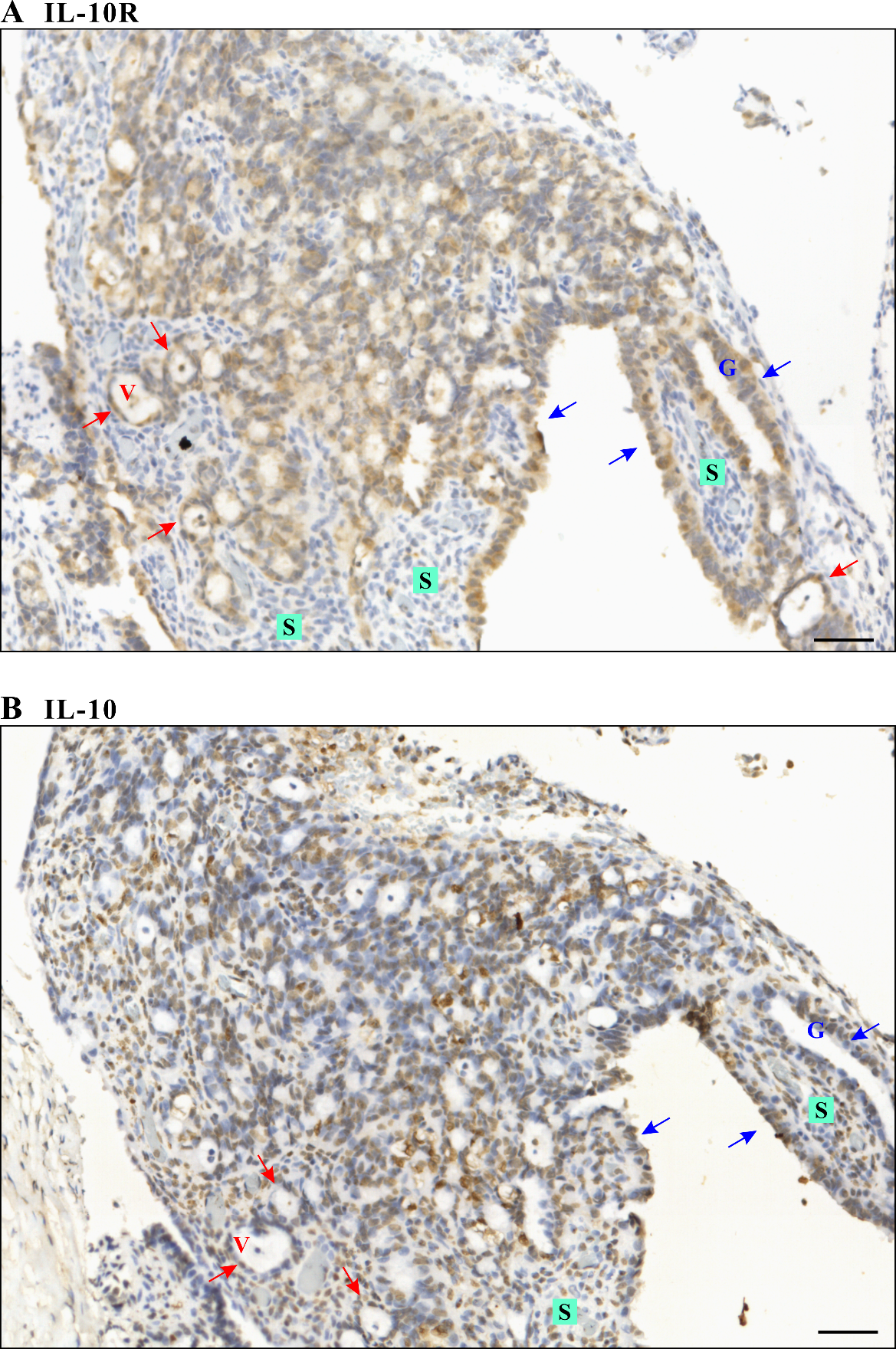


**Figure S7.** The IL-10R or IL-10-expressing non-immune cell types in human endometrioma. Enlarged images of (A) IL-10R or (B) IL-10 expression (brown colour) in ovarian endometrioma tissues as shown in Figure 5A. Red arrows indicate blood vessels and blue arrows glandular epitheliums. S, stroma. Blue, haematoxylin counterstain. Scale bars = 50 μm.


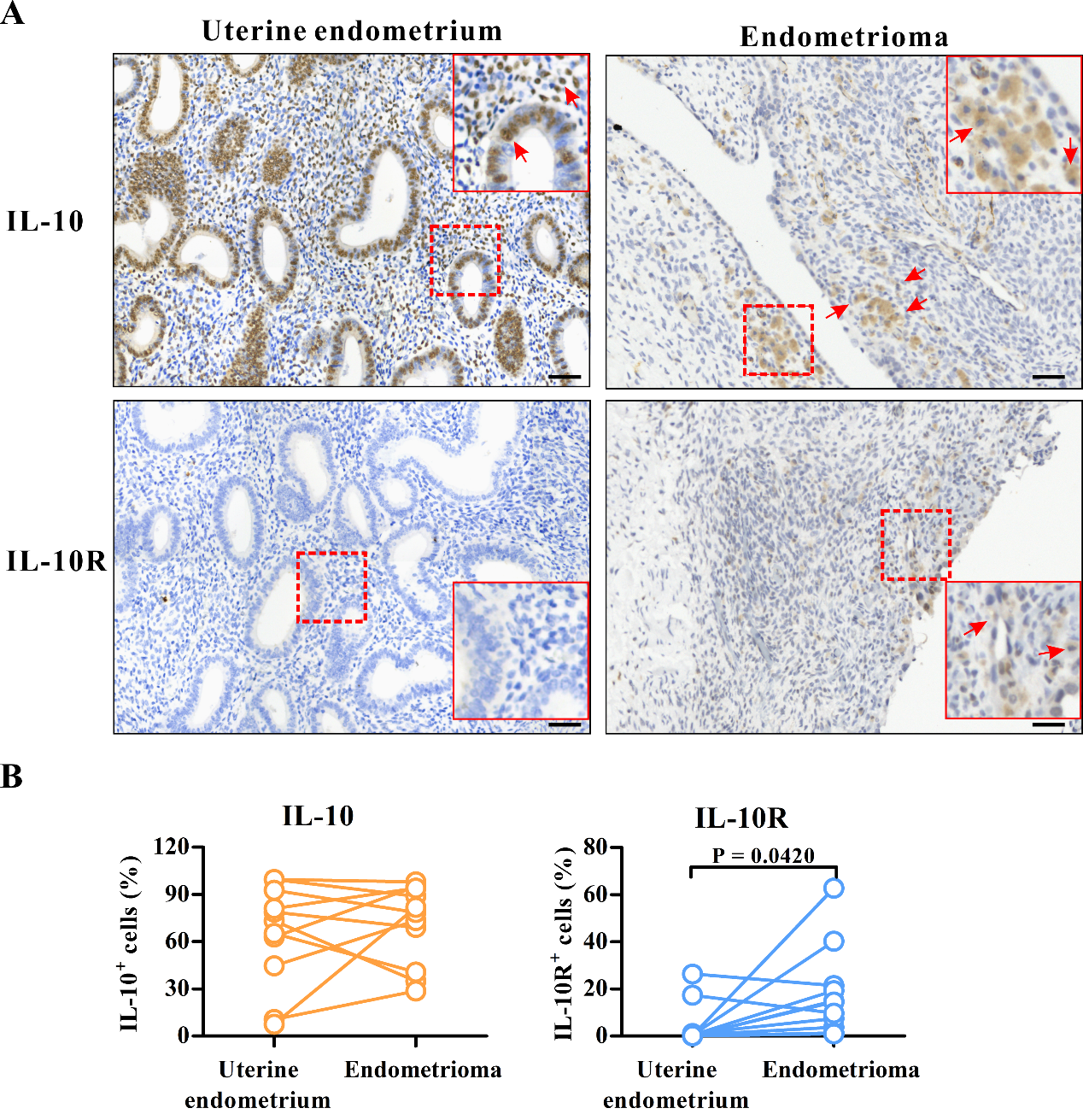


**Figure S8.** The expression of IL-10 or IL-10R in normal uterine endometrium and human endometrioma tissues. (A) Immunohistochemistry for IL-10 or IL-10R (brown colour) in a representative endometrioma lesion as well as the matched normal endometrium. The insets (solid box) show a two-fold enlarged image from the dashed box. Arrows indicate positive cells. Blue, haematoxylin counterstain. Scale bars = 50 μm. (B) Quantification of positive cell frequencies in each endometrioma and corresponding uterine endometrium analysed by HistoQuest software. Number of endometrioma samples, 11 for both markers; number of uterine endometrial samples, 10 for both markers. *p* < 0.05 was considered significant by the Kruskal–Wallis test and then Dunn’s multiple comparison test.

**Table S1.** Demographic characteristics of the patients with endometriosis

| **Characteristic** | **Patients with endometrioma (*n*** **=** **10)** |
| --- | --- |
| Age (years)* | 39.5 ± 5.3 |
| BMI (kg/m^2^)* | 22.71 ± 3.47 |
| Stage of endometriosis |  |
| Stage I or II | *n* = 0 |
| Stage III or IV | *n* = 10 |

*Data are the mean ± SD.

BMI, body mass index.
